# RNA-seq profiling of white and brown adipocyte differentiation treated with epigallocatechin gallate

**DOI:** 10.1038/s41597-022-01149-0

**Published:** 2022-02-08

**Authors:** Pengpeng Zhang, Wei Wu, Chunyu Du, Xiang Ji, Yaling Wang, Qiu Han, Hiaxia Xu, Cencen Li, Yongjie Xu

**Affiliations:** 1 Department of Biotechnology, College of Life Sciences, Xinyang Normal University, Xinyang, China; 2 Institute for Conservation and Utilization of Agro-Bioresources in Dabie Mountains, Xinyang Normal University, Xinyang, China

**Keywords:** Diseases, Cell biology

## Abstract

Due to serious adverse effects, many of the approved anti-obesity medicines have been withdrawn, and the selection of safer natural ingredients is of great interest. Epigallocatechin gallate (EGCG) is one of the major green tea catechins, and has been demonstrated to possess an anti-obesity function by regulating both white and brown adipose tissue activity. However, there are currently no publicly available studies describing the effects of EGCG on the two distinct adipose tissue transcriptomes. The stromal vascular fraction (SVF) cell derived from adipose tissue is a classic cell model for studying adipogenesis and fat accumulation. In the current study, primary WAT and BAT SVF cells were isolated and induced to adipogenic differentiation in the presence or absence of EGCG. RNA-seq was used to determine genes regulated by EGCG and identify the key differences between the two functionally distinct adipose tissues. Taken together, we provide detailed stage- and tissue-specific gene expression profiles affected by EGCG. These data will be valuable for obesity-related clinical/basic research.

## Abstract (summary)

**Table:** 

Measurement(s)	white adipose tissue transcriptome • brown adipose tissue transcriptome
Technology Type(s)	RNA sequencing
Factor Type(s)	epigallocatechin gallate presence

Machine-accessible metadata file describing the reported data: 10.6084/m9.figshare.19067663

## Background & Summary

Obesity is defined as abnormally excessive body fat accumulation. It has been accepted as one of the global health issues because it is highly associated with many diseases such as diabetes, dyslipidemia, and cardiovascular diseases^1^, which are harmful to health and lead to significant economic and social burdens in many countries worldwide.

The main feature of obesity is enlarged adipose tissue, which is dependent on hyperplasia (formation of new adipocytes from precursor cells) and hypertrophy of pre-existing adipocytes^2^. Animal adipose tissue mainly includes white adipose tissue (WAT) and brown adipose tissue (BAT), which could be distinguished from cell structures and functional roles. Many large lipid droplets accumulate in WAT, while only a few small lipid droplets can be identified in BAT. Whereas WAT stores energy as triglycerides, BAT generates heat through fatty acid metabolism^3^. Therefore, strategies that regulate WAT adipocyte size, number, or stimulation of BAT are considered a possible therapeutic approach for combating obesity^4^.

Medical approaches to the prevention and treatment of obesity include anti-obesity drugs and surgery. Because surgery is limited to severely obese individuals, anti-obesity drugs are a promising solution to obesity. However, many adverse drug reactions or side effects have raised public concern^5^. Hence, the selection of natural products with high anti-obesity efficacy, safety, and long-term effects is receiving more attention.

Recently, the anti-obesity effects of green tea have been greatly investigated. Green tea catechins are the predominant form of flavor, which can be extracted from green tea. It has been reported that green tea catechins, especially epigallocatechin gallate (EGCG) could decrease fat accumulation^6^. Evidence from *in vitro* and *in vivo* studies indicates that EGCG plays role in various nutritional and pharmacological functions, including anti-inflammatory^7^, antioxidative^8^, antiangiogenesis^9^, and cardiovascular protection effects^10^. Recent studies also demonstrated that EGCG regulates both WAT and BAT activity. It could not only reduce white adipose tissue mass by inhibiting the synthesis of *de novo* fatty acids and increasing lipolysis^11^, but also increase thermogenesis and mitochondrial biogenesis in brown adipose tissues^12^. However, a systemic comparison of transcriptomes that are affected by EGCG in both WAT and BAT is lacking, which is of great interest to the field.

**Fig. 1:**
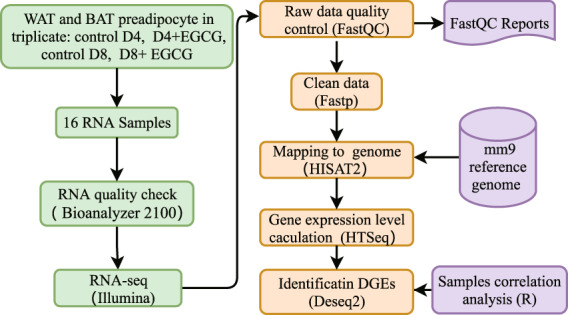
The workflow of data collection and technical analysis for this study.

To address this question, primary WAT and BAT preadipocytes were isolated and induced adipogenic differentiation in the presence or absence of EGCG. Samples were collected on Day 4 and Day 8 post differentiation, which represented the early and mature stages of adipogenesis, respectively. Then, we performed Illumina RNA-seq and bioinformatics analysis of the mRNA profile (Fig. 1). This comprehensive dataset will provide the stage- and tissue-specific effects of EGCG on adipogenesis and fat storage. The dataset might be a very useful resource to researchers for the development of therapies to treat obesity and other metabolic diseases.

## Methods

### Cell culture

All procedures involving mice were approved by the Xinyang Normal University Animal Care and Use Committee. Primary WAT and BAT stromal vascular fraction (SVF) cells were isolated as previously described^13,14^. Briefly, inguinal WAT and interscapular BAT were obtained from 6-week-old mice on a C57BL/6 J background. Fat pads were cut into small pieces and incubated with collagenase digestion solution (1.5 mg ml^−1^, #SCR103, Sigma-Aldrich). WAT was incubated for 30 min and BAT was incubated for 50 min. Then, an equal volume of growth medium (DMEM containing 20% FBS) was added to terminate digestion. To remove tissue debris, the digestion was filtered through 100-μm and 70-μm cell trainers. Next, it was centrifuged at 450 g for 8 min. Then, the SVF cells pellet was resuspended and cultured in growth medium, at 37 °C with 5% CO_2_. When the cells reached 90% confluence, the growth medium was changed to DMEM containing 10% FBS, and the cells were induced to adipogenic differentiation by supplementation with a cocktail containing 2.85 mM recombinant human insulin (#I8830, Solarbio), 0.3 mM dexamethasone (#D8040, Solarbio), and 0.63 mM 3-isobutyl-methylxanthine (#I7018, Sigma-Aldrich). After four days, the cocktail was changed to 200 nM insulin, and 10 nM T3 (#T6397, Sigma-Aldrich) to induce mature adipocytes. EGCG (5 μM) or DMSO control (1:1000) was added to the induction medium and differentiation medium from Day 4 to 8 of adipogenesis. The medium was changed every 2 days. To examine the lipid droplets, oil red O staining was performed. Briefly, the mature adipocytes were washed twice with PBS. Then, the cells were fixed with 10% formaldehyde for 5 min. Next, the cells were stained with Oil red O staining solutions (#G1262, Solarbio) for 30 min. After staining, the images were captured with an Axio Observer 3 Zeiss microscope (Carl Zeiss, Germany).

### RNA reparation

TRIzol (#15596026, Thermo Fisher Scientific) was used to collect RNA. RNA concentration was measured using a Qubit® RNA Assay Kit in a Qubit® 2.0 Flurometer (Life Technologies, USA). A Bioanalyzer 2100 system (Agilent Technologies, USA) was used to examine RNA integrity.

### Real-time PCR analysis

Real-time PCR was performed as previously described^15^. Briefly, Reverse Transcription Kit (#RR037A, Takara) was used to synthesize cDNA. Real-time PCR was performed on a CFX96 Real-Time System (Bio-Rad Laboratories, Singapore) according to the manufacture’s instructions. 18 S was used as housekeeping genes. The (2^−ΔΔCt^) method was used to calculate the gene expression levels.

### RNA-Seq library construction, RNA-sequencing, and bioinformatics analysis

The sequencing library was built using the NEBNext® UltraTM RNA Library Prep Kit for Illumina® (#E7530L, NEB), and 3 μg RNA for each sample was used. Clustering of the index-coded samples was performed on a cBot Cluster Generation System using TruSeq PE Cluster Kit v3-cBot-HS (#PE-401-3001, Illumina) according to the manufacturer’s instructions. Sequencing was carried out on an Illumina HIseq 4, 000 platform. 150-bp paired-end reads were generated.

### Bioinformatics analyses

Quality control of sequences was examined with FastQC (version 0.11.9)^16^. Clean data were obtained using Fastp (version 0.20.1)^17^ to remove low-quality reads, read adapters, and reads containing poly-N. Then, the clean data were subjected to mapping with the Mus musculus mm9 genome reference by HISAT2 (version 2.2.0)^18^. The average input read count was 44.91 million per sample (range 20.12 million to 63.9 million), and the average percentage of uniquely aligned reads was 90.70% (range 87.57% to 92.5%). The read counts for each gene were calculated by HTSeq (version 0.11)^19^. The third biological replication was sequenced at different baches as the other biological replications. The expression levels were corrected for batch effects using the Combat_Seq function from the SVA R package (version 3.42.0)^20^. Principal component analysis (PCA) was generated with FactoMineR^21^ to assess variance between sample groups and sample replicates. Differential gene expression was identified by DESeq2 (version 1.10.1). A heatmap was generated with pheatmap (version 1.0.12)^22^ to show the differentially expressed genes between the control and EGCG-treated group. The R package ggplot2 (version 3.3.4)^23^ was used to generate volcano plots and compare the gene expression levels between the control and EGCG-treated groups.

## Data Records

The RNA-Seq data were deposited in the NCBI Sequence Read Archive (SRA) under the accession number SRP318155^24^. The metadata records regarding the sample’s information and RNA-seq read statistics are provided in Tables 1 and 2, respectively.

**Table 1:** RNA-seq samples information.

Subject	Source	Treatment	Sample Name	Protocol 1	Protocol 2	GEO Accession
mouse WAT replication 1	differentiation day 4	Control	mW41	RNA extraction	RNA-seq	GSE173710
mouse WAT replication 2	differentiation day 4	Control	mW42	RNA extraction	RNA-seq	GSE173710
mouse WAT replication 3	differentiation day 4	Control	mW43	RNA extraction	RNA-seq	GSE173710
mouse WAT replication 1	differentiation day 4	Control	mW81	RNA extraction	RNA-seq	GSE173710
mouse WAT replication 2	differentiation day 4	Control	mW82	RNA extraction	RNA-seq	GSE173710
mouse WAT replication 3	differentiation day 4	Control	mW83	RNA extraction	RNA-seq	GSE173710
mouse WAT replication 1	differentiation day 8	EGCG	mWE41	RNA extraction	RNA-seq	GSE173710
mouse WAT replication 2	differentiation day 8	EGCG	mWE42	RNA extraction	RNA-seq	GSE173710
mouse WAT replication 3	differentiation day 8	EGCG	mWE43	RNA extraction	RNA-seq	GSE173710
mouse WAT replication 1	differentiation day 8	EGCG	mWE81	RNA extraction	RNA-seq	GSE173710
mouse WAT replication 2	differentiation day 8	EGCG	mWE82	RNA extraction	RNA-seq	GSE173710
mouse WAT replication 3	differentiation day 8	EGCG	mWE83	RNA extraction	RNA-seq	GSE173710
mouse BAT replication 1	differentiation day 4	Control	mB41	RNA extraction	RNA-seq	GSE173710
mouse BAT replication 2	differentiation day 4	Control	mB42	RNA extraction	RNA-seq	GSE173710
mouse BAT replication 3	differentiation day 4	Control	mB43	RNA extraction	RNA-seq	GSE173710
mouse BAT replication 1	differentiation day 4	Control	mB81	RNA extraction	RNA-seq	GSE173710
mouse BAT replication 2	differentiation day 4	Control	mB82	RNA extraction	RNA-seq	GSE173710
mouse BAT replication 3	differentiation day 4	Control	mB83	RNA extraction	RNA-seq	GSE173710
mouse BAT replication 1	differentiation day 8	EGCG	mBE41	RNA extraction	RNA-seq	GSE173710
mouse BAT replication 2	differentiation day 8	EGCG	mBE42	RNA extraction	RNA-seq	GSE173710
mouse BAT replication 3	differentiation day 8	EGCG	mBE43	RNA extraction	RNA-seq	GSE173710
mouse BAT replication 1	differentiation day 8	EGCG	mBE81	RNA extraction	RNA-seq	GSE173710
mouse BAT replication 2	differentiation day 8	EGCG	mBE82	RNA extraction	RNA-seq	GSE173710
mouse BAT replication 3	differentiation day 8	EGCG	mBE83	RNA extraction	RNA-seq	GSE173710

**Table 2:** RNA-seq reads information.

Sample Name	Sequencing Strategy	Raw reads number	Clean reads number	Q20	Q30	Mapping rate (%)
mW41	PE150	63353952	62659858	98.05	94.45	91.20
mW42	PE150	53454310	52794832	98.04	94.44	91.38
mW43	PE150	21568205	20498381	98.06	94.71	88.61
mW81	PE150	56009120	55212996	98.00	94.31	90.98
mW82	PE150	59735598	58768178	98.04	94.42	91.40
mW83	PE150	21857011	20971455	97.97	94.44	88.46
mWE41	PE150	61953094	60984426	97.81	93.88	91.57
mWE42	PE150	49808446	49100258	98.12	94.63	91.49
mWE43	PE150	31188130	29905593	97.97	94.45	88.54
mWE81	PE150	64305198	63472412	97.93	94.13	91.92
mWE82	PE150	49264074	48649180	98.01	94.35	91.92
mWE83	PE150	21438213	20127271	97.99	94.56	88.99
mB41	PE150	50653710	49932582	98.03	94.37	91.83
mB42	PE150	56322254	55589762	98.08	94.51	91.81
mB43	PE150	23036272	21796117	97.98	94.52	87.57
mB81	PE150	55074040	54072312	97.99	94.29	92.04
mB82	PE150	61684398	60751782	97.93	94.13	91.92
mB83	PE150	23510964	22462259	98.03	94.58	88.10
mBE41	PE150	58047352	57059452	98.16	94.68	92.07
mBE42	PE150	48561368	47888428	98.08	94.51	91.97
mBE43	PE150	24374910	23127966	98.03	94.61	88.80
mBE81	PE150	57668480	56867060	98.07	94.51	92.47
mBE82	PE150	64849394	63939362	98.01	94.37	92.47
mBE83	PE150	22612540	21115135	97.99	94.56	89.24

## Technical Validation

### Quality control of cell differentiation

**Fig. 2:**
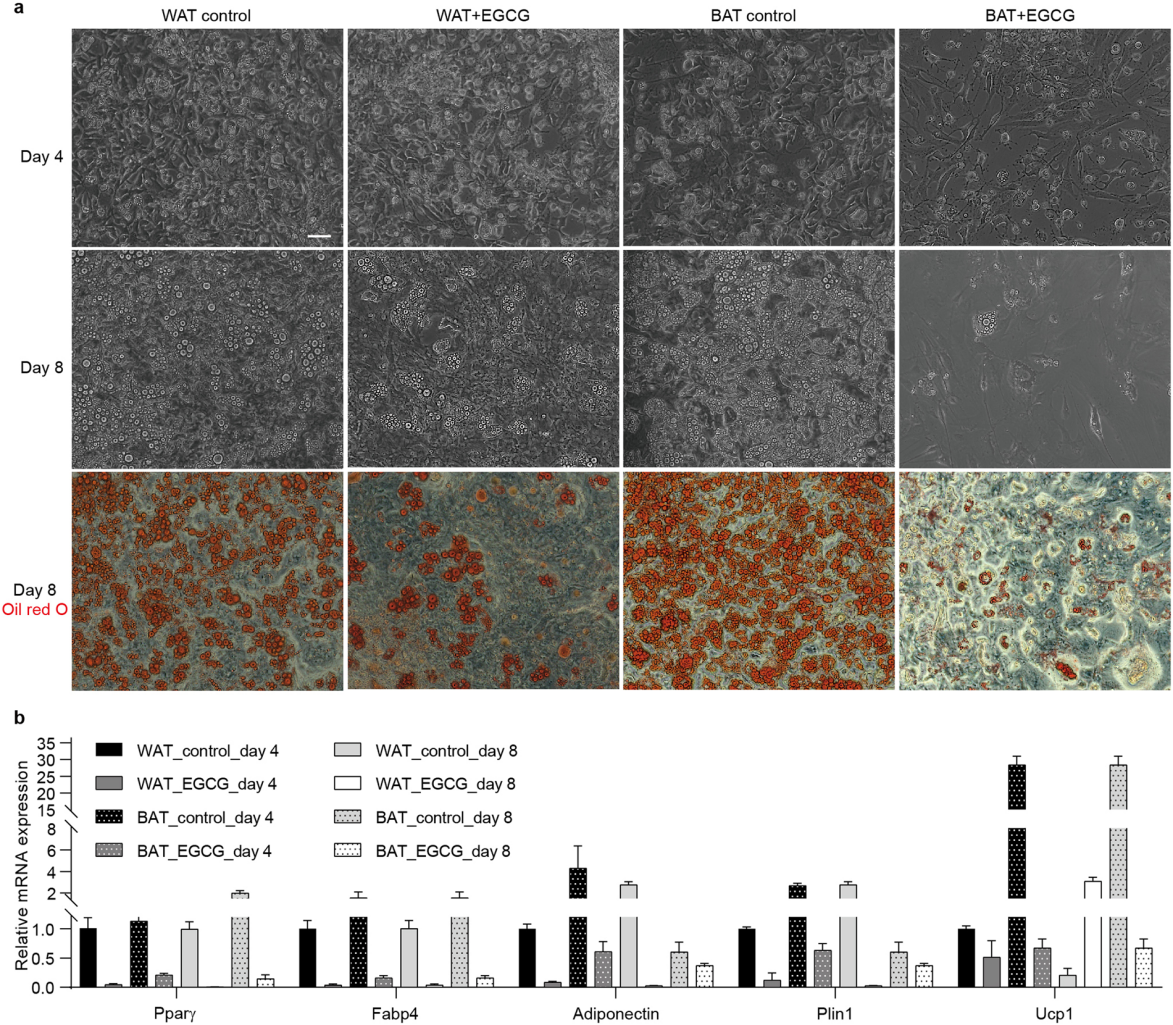
Differentiation of WAT and BAT SVF cells. (**a**) Representative pictures of the cells at Day 4 and Day 8 post differentiation. Oil red O staining was performed to visualize lipid droplets at Day 8 post differentiation. Scale bar = 20 µm. (**b**) Relative expression of the selected white and brown adipocyte marker genes (n = 3). Mean and standard deviations are shown.

The quality of WAT and BAT SVF adipogenic differentiation was examined at Day 4 and Day 8 post differentiation. As shown in Fig. 2a, the cells underwent a morphology change, from spindle to round and small droplets began to accumulate at Day 4 in the control groups. Furthermore, many more large droplets accumulated at Day 8 post differentiation. Oil red O staining showed that both WAT and BAT SVF cells differentiated well at Day 8. Compared to the control groups, the SVF cells treated with EGCG showed fewer lipid droplets, which indicated that EGCG inhibits SVF cells adipogenic differentiation. Consistent with these findings, real-time PCR analysis showed that adipose marker genes (*Pparγ*, *Fabp4*, *Adiponectin*, and *Plin1*) were expressed at much higher levels in the control groups than in the EGCG treated groups. In addition, the BAT SVF cells in the control groups expressed approximately 30 times more *Ucp1* (BAT marker gene) than the WAT SVF (Fig. 2b). The above results suggested that SVF cells in the control groups differentiate into the right subtype of adipocytes and that EGCG inhibits SVF cell adipogenic differentiation.

### Quality control of RNA integrity

The quality of total RNA was examined by an Agilent Bioanalyzer 2100. All of the samples showed high RNA integrity (RIN value ranging from 7.7 to 10) and could be used for downstream sequencing.

### RNA-Seq data quality

**Fig. 3:**
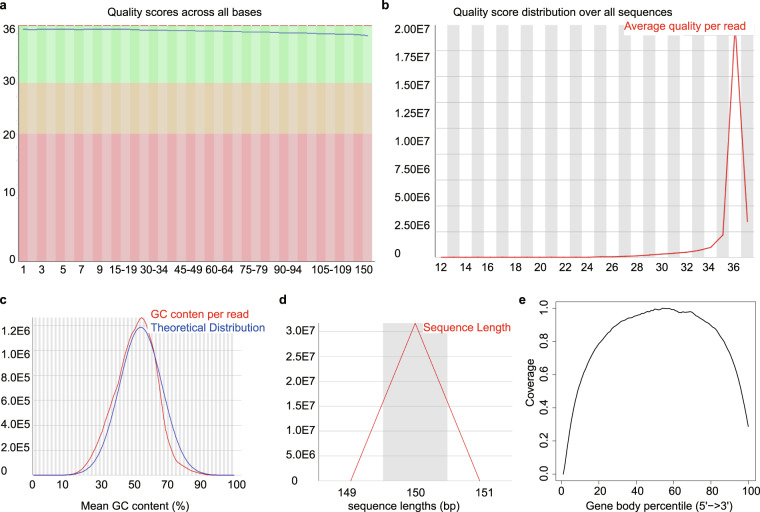
Representative quality check of RNA-seq. (**a**) Representative quality score distribution for all 150 bp bases. (**b**) Representative quality score distribution of all sequences. (**c**) Representative distribution of GC content for each sequence. (**d**) Representative distribution of sequence length. (**e**) Coverage uniformity along with transcripts.

**Fig. 4:**
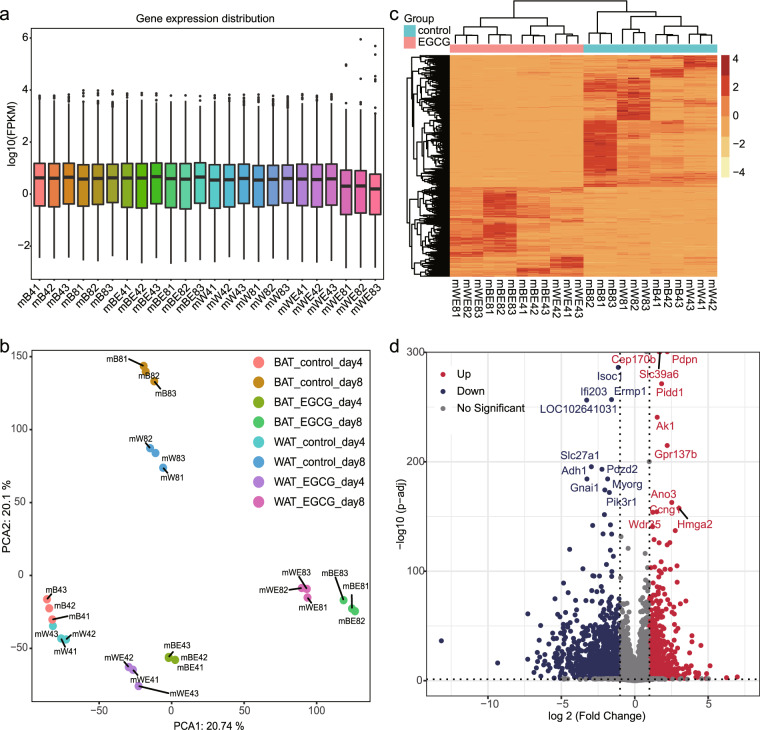
(**a**) Box plot showing the expression level of mRNA in all samples. (**b**) Principal component analysis results. (**c**) Heatmap showing differentially expressed genes between EGCG treated and control cells. (**d**) Volcano plot comparing gene expression levels between EGCG treated and control cells. Red indicates significantly upregulated genes, while blue indicates significantly downregulated genes. The names of the top 20 significantly regulated genes arranged by p-adj are labeled.

The raw RNA-seq data quality was determined by FastQC. A representative FastQC report is depicted in Fig. 3. As indicated, the reads had universally high-quality values(Fig. 3a,b). The distribution of GC content was similar to the theoretical distribution, which indicates that the samples were free from contamination (Fig. 3c). Meanwhile, the sequence length distribution showed a peak only at 150 bp, which corresponded to the fragment sizes of the RNA-seq libraries (Fig. 3d). Then, the geneBodyCoverage.py script from the RseQC package was used to assess the quality of the reads and no significant 5′ or 3′ end bias was identified (Fig. 3e). In addition, all other fastqc files showed similar reports and were qualified for downstream analysis. Subsequently, a very high percentage (more than 87.57%) of reads were mapped to the reference genome mm9 (Table 2). Next, the genes expression levels were examined. The box plot showed that the global expression levels of mRNA in all samples were similar (Fig. 4a). Furthermore, analysis of all the expressed genes by PCA plot showed that samples derived from a different tissue, time points, and treatment fell into distinct groups, suggesting variability between groups. Meanwhile, the biological replicates settled near each other, indicating high repeatability (Fig. 4b).

### Identification of genes affected by EGCG

To identify the genes affected by EGCG, gene expression levels of the control and EGCG-treated groups were analyzed by DESeq2. The threshold criteria were set as p-adj < 0.01 and absolute (log _2_ FoldChange) > 1. The genes that met these criteria were further analyzed with a heatmap and volcano map. Figure 4c shows that the samples from the control groups and the EGCG-treated groups fell into two separate groups, indicating that EGCG robustly affects gene expression during adipogenesis. In addition, samples from triplicate replication were densely clustered, indicating high repeatability. Figure 4d shows that the EGCG regulated the expression of many genes, and the top significantly differentially expressed genes ordered by p-adj were *Cep170b*, *Pdpn*, *Soc1*, *Slc39a6*, and *Pidd1*.

## Usage Notes

RNA-seq has been widely used to study gene expression in recent decades. It is a powerful method to systematically determine the molecular pathways affected by bioactive substances. Recently, EGCG has been recognized as one of the natural products with high anti-obesity efficacy. The data could provide important findings of the genes regulated by EGCG in adipogenesis and provide information about the role of EGCG in lipid accumulation and fat metabolism.

One major advantage of the data is that detailed stage- and tissue-specific gene expression profiles affected by EGCG were investigated. The data may be valuable to obesity-related clinical/basic research. Of note, RNA from the three replications was collected simultaneously, but the third replication was not sequenced at the same time as the other two. Although the quality of all RNA met the requirements of RNA-seq, it would be beneficial to correct the batch effect.

## Data Availability

In the current study, the following open access software was used as described in the Methods section. For all the software, we used default parameters, and no custom code was used beyond the tools listed. 1. FastQC (version 0.11.9) was used to check the quality of raw FASTQ sequencing data: http://www.bioinformatics.babraham.ac.uk/projects/fastqc/. 2. Fastp (version 0.20.1) was used to trim adapters and filter quality reads: https://github.com/OpenGene/fastp. 3. HISAT2 (version 2.2.0) was used to map sequence reads to the mouse mm9 genome: http://daehwankimlab.github.io/hisat2/. 4. DESeq2 (version 1.10.1) was used to identify differentially expressed genes: https://bioconductor.org/packages/release/bioc/html/DESeq2.html. 5. FactoMineR (version 2.4) was used to perform PCA: https://cran.r-project.org/web/packages/FactoMineR/index.html. 6. Pheatmap (Version 1.0.12) was used to plot the heatmap: https://cran.r-project.org/web/packages/pheatmap/. 7. Ggplot2 (version 3.3.4) was used to generate the volcano plot: https://cran.r-project.org/web/packages/ggplot2/index.html. 8. The Combat_Seq function from R package SVA was used to correct the batch effect of samples in each batch: https://bioconductor.org/packages/release/bioc/html/sva.html. 9. The GeneBodyCoverage.py script from RseQC package (version 4.0.0) was used to evaluate the quality of the reads: https://sourceforge.net/projects/rseqc/files/.

## References

[1] Blüher M (2019). Obesity: global epidemiology and pathogenesis. Nat. Rev. Endocrinol..

[2] Stefan N (2020). Causes, consequences, and treatment of metabolically unhealthy fat distribution. Lancet Diabetes Endocrinol.

[3] Ikeda K, Maretich P, Kajimura S (2018). The Common and Distinct Features of Brown and Beige Adipocytes. Trends Endocrinol. Metab..

[4] Maurer, S., Harms, M. & Boucher, J. The colorful versatility of adipocytes: white‐to‐brown transdifferentiation and its therapeutic potential in humans. *FEBS J* (2020). 10.1111/febs.15470 32621398

[5] Ammori B (2020). Medical and surgical management of obesity and diabetes: what’s new?. Diabet. Med..

[6] Martel J (2016). Anti-obesogenic and antidiabetic effects of plants and mushrooms. Nat. Rev. Endocrinol.

[7] Cavet ME, Harrington KL, Vollmer TR, Ward KW, Zhang J-Z (2011). Anti-inflammatory and anti-oxidative effects of the green tea polyphenol epigallocatechin gallate in human corneal epithelial cells. Mol. Vis..

[8] Gao Z (2016). Targeting HO-1 by epigallocatechin-3-gallate reduces contrast-induced renal injury via anti-oxidative stress and anti-inflammation pathways. PLoS ONE.

[9] Wang J, Man GCW, Chan TH, Kwong J, Wang CC (2018). A prodrug of green tea polyphenol (–)-epigallocatechin-3-gallate (Pro-EGCG) serves as a novel angiogenesis inhibitor in endometrial cancer. Cancer Lett..

[10] Wolfram S (2007). Effects of green tea and EGCG on cardiovascular and metabolic health. J. Am. Coll. Nutr..

[11] Li F (2018). EGCG Reduces Obesity and White Adipose Tissue Gain Partly Through AMPK Activation in Mice. Front Pharmacol.

[12] Lee MS, Shin Y, Jung S, Kim Y (2017). Effects of epigallocatechin-3-gallate on thermogenesis and mitochondrial biogenesis in brown adipose tissues of diet-induced obese mice. Food Nutr Res.

[13] Zhang, P.-p. *et al*. Identification of Circular RNA Expression Profiles in White Adipocytes and Their Roles in Adipogenesis. *Front Physiol* **12**, 10.3389/fphys.2021.728208 (2021). 10.3389/fphys.2021.728208 PMC8417237 34489740

[14] Zhang, P. *et al*. Assessment of CircRNA Expression Profiles and Potential Functions in Brown Adipogenesis. *Front Genet* **12**, 10.3389/fgene.2021.769690 (2021). 10.3389/fgene.2021.769690 PMC8569449 34745232

[15] Shan T (2016). Adipocyte-specific deletion of mTOR inhibits adipose tissue development and causes insulin resistance in mice. Diabetologia.

[16] FastQC. FastQC: a quality control tool for high throughput sequence data. (2016).

[17] Chen S, Zhou Y, Chen Y, Gu J (2018). fastp: an ultra-fast all-in-one FASTQ preprocessor. Bioinformatics (Oxford, England).

[18] Kim D, Paggi JM, Park C, Bennett C, Salzberg SL (2019). Graph-based genome alignment and genotyping with HISAT2 and HISAT-genotype. Nat. Biotechnol..

[19] Anders S, Pyl PT, Huber W (2015). HTSeq—a Python framework to work with high-throughput sequencing data. Bioinformatics.

[20] Zhang Y, Parmigiani G, Johnson WE (2020). ComBat-seq: batch effect adjustment for RNA-seq count data. NAR Genom Bioinform.

[21] Lê S, Josse J, Husson F (2008). FactoMineR: an R package for multivariate analysis. J Stat Softw.

[22] Kolde R, Kolde MR (2015). Package ‘pheatmap’. R package.

[23] Wickham H (2011). ggplot2. Wiley Interdiscip Rev Comput Stat.

[24] Zhang P, Xu Y, Xu H, Li C (2021). NCBI Sequence Read Archive.

